# Nonmalignant Respiratory Effects of Chronic Arsenic Exposure from Drinking Water among Never-Smokers in Bangladesh

**DOI:** 10.1289/ehp.9507

**Published:** 2007-11-06

**Authors:** Faruque Parvez, Yu Chen, Paul W. Brandt-Rauf, Alfred Bernard, Xavier Dumont, Vesna Slavkovich, Maria Argos, Jeanine D’Armiento, Robert Foronjy, M. Rashidul Hasan, HEM Mahbubul Eunus, Joseph H. Graziano, Habibul Ahsan

**Affiliations:** 1 Department of Environmental Health Sciences, Mailman School of Public Health, Columbia University, New York, USA; 2 Department of Environmental Medicine, New York University School of Medicine, New York, USA; 3 Department of Public Health, Catholic University of Louvain, Brussels, Belgium; 4 Department of Epidemiology, Mailman School of Public Health, Columbia University, New York, USA; 5 Department of Medicine, College of Physicians and Surgeons, Columbia University, New York, USA; 6 Chest Institute, Dhaka, Bangladesh; 7 Columbia University Arsenic Research Project in Bangladesh, Dhaka, Bangladesh; 8 Department of Health Studies and Cancer Research Center, University of Chicago, Chicago, USA

**Keywords:** arsenic, Bangladesh, CC16, Clara cell 16, drinking water, epithelial lung damage, respiratory illnesses

## Abstract

**Background:**

Arsenic from drinking water has been associated with malignant and nonmalignant respiratory illnesses. The association with nonmalignant respiratory illnesses has not been well established because the assessments of respiratory symptoms may be influenced by recall bias or interviewer bias because participants had visible skin lesions.

**Objectives:**

We examined the relationship of the serum level of Clara cell protein CC16—a novel biomarker for respiratory illnesses—with well As, total urinary As, and urinary As methylation indices.

**Methods:**

We conducted a cross-sectional study in nonsmoking individuals (*n* = 241) selected from a large cohort with a wide range of As exposure (0.1–761 μg/L) from drinking water in Bangladesh. Total urinary As, urinary As metabolites, and serum CC16 were measured in urine and serum samples collected at baseline of the parent cohort study.

**Results:**

We observed an inverse association between urinary As and serum CC16 among persons with skin lesions (β = −0.13, *p* = 0.01). We also observed a positive association between secondary methylation index in urinary As and CC16 levels (β = 0.12, *p* = 0.05) in the overall study population; the association was stronger among people without skin lesions (β = 0.18, *p* = 0.04), indicating that increased methylation capability may be protective against As-induced respiratory damage. In a subsample of study participants undergoing spirometric measures (*n* = 31), we observed inverse associations between urinary As and predictive FEV_1_ (forced expiratory volume measured in 1 sec) (*r* = −0.37; FEV_1_/forced vital capacity ratio and primary methylation index (*r* = −0.42, *p* = 0.01).

**Conclusions:**

The findings suggest that serum CC16 may be a useful biomarker of epithelial lung damage in individuals with arsenical skin lesions. Also, we observed the deleterious respiratory effects of As exposure at concentrations lower than reported in earlier studies.

Although several international studies have shown the effects of arsenic on elevated lung cancer risk, there has been relatively little research on its role in nonmalignant respiratory illnesses (Chiou et al. 1995; Mazumder et al. 1998; Smith et al. 1998). Studies from India (Mazumder et al. 1998), Bangladesh (Milton et al. 2003; Milton and Rahman 2002), and Chile (Smith et al. 1998) have reported a high prevalence of respiratory symptoms among As-exposed individuals; these studies were largely limited to people exposed to high As concentrations who had visible arsenical skin lesions. Six studies from India and Bangladesh showed a high prevalence of respiratory symptoms among people exposed to water As concentrations > 500 μg/L and with visible arsenical lesions (De et al. 2004; Mazumder et al. 2000, 2005; Milton et al. 2003; Milton and Rahman 2002; von Ehrenstein et al. 2005); three of these studies were conducted in the same source population in India (Mazumder et al. 2000, 2005; von Ehrenstein et al. 2005). The first study (Mazumder et al. 2000), which had a large sample size (*n* = 6,864), reported 5–23 times higher prevalence of common respiratory symptoms (e.g., chronic cough, abnormal chest sound, shortness of breath) among people with arsenical skin lesions. The second study (von Ehrenstein et al. 2005), with a smaller sample size (*n* = 287), reported a higher risk [odds ratios (ORs), 2.8–3.8] of common respiratory symptoms and lower FEV_1_ (forced expiratory volume measured in 1 sec) and FVC (forced vital capacity) among people with arsenical lesions. In their most recent study (*n* = 258), Mazumder et al. (2005) reported a 10-fold increased risk of chronic obstructive pulmonary disease (COPD) among people with arsenical skin lesions. In an earlier study from India in 156 As-exposed (> 600 μg/L) individuals with skin lesions, De et al. (2004) reported that 57% of subjects had respiratory symptoms and 53% had restrictive lung disease. The authors also found that 41% of the study participants had both obstructive and restrictive lung diseases. Two studies from neighboring Bangladesh have also reported an elevated risk of respiratory symptoms such as chronic cough [risk ratio (RR) = 2.1] and chronic bronchitis (RR = 2.68) associated with As exposure in people with visible skin lesions (Milton et al. 2003; Milton and Rahman 2002).

In a study in an As-endemic area of Chile, Smith et al. (2006) found a high mortality from COPD due to arsenic. Two other studies from Chile also reported a high prevalence of respiratory illness among children with arsenical skin lesions compared with those without such lesions (Zaldivar 1980; Zaldivar and Ghani 1980). In addition, in an intervention study, Borgono et al. (1977) observed a reduction in prevalence (from 23% to 7%) of common respiratory illnesses after providing As-free water, reinforcing the effect of As on chronic respiratory illness.

In almost all of the studies published to date, assessments of respiratory illnesses are problematic for two reasons. First*,* most of these studies were conducted among people with visible arsenical lesions that may have biased the assessment of respiratory symptoms in the study participants (Mazumder et al. 2000). Second, respiratory symptoms were assessed either by self-report, which may be affected by recall bias, or by lung function tests, which require patient cooperation and may be subject to information bias, leading to either over- or underestimation of the true measure of association. For instance, if persons with skin lesions or high As exposure are more cooperative or if they receive more attention, the measure of association would be, to some extent, overestimated. However, detection of respiratory illness can be improved and bias can be avoided by using valid biomarkers. In the present study, we examined the serum level of Clara cell protein CC16, a novel biomarker for detecting respiratory illnesses, in 241 nonsmokers chronically exposed to wide levels of As from drinking water. Although the clinical significance of early epithelial changes detected by serum CC16 remains to be fully determined, several studies have shown that CC16 can be used as a biomarker for detecting respiratory effects induced by environmental exposures such as air pollution and tobacco smoking (Bernard et al. 1994; Berthoin et al. 2004; Broeckaert and Bernard 2000; Broeckaert et al. 2000; Johansson et al. 2005; Lagerkvist et al. 2004). The objective of our analyses was to examine the effects of As exposure on lung injury using the serum level of CC16 and several indices of As exposure.

## Methods

### Selection of study participants

The data we present here are from a subset of participants of a large ongoing prospective cohort study in Araihazar, Bangladesh. The goals of the parent multidisciplinary epidemiologic investigation are to examine the health effects of As exposure from drinking water in order to guide policy. A detailed description of the parent cohort study has been published elsewhere (Ahsan et al. 2006a). In short, 11,746 adults who had been drinking As-contaminated water at a broad range of As concentrations for at least 3 years were recruited between October 2000 and May 2002 and have since been followed at 2-year intervals. Demographic and smoking data and water, urine, and blood samples were collected at the baseline and at follow-up visits. At each visit, As-induced skin lesion status was evaluated, quantified, and validated by our study physicians and expert dermatologists (Ahsan et al. 2006a). As-related skin lesions are known to be a hallmark of chronic As poisoning. These lesions include discoloration of skin with pigmentation and, in many cases, are accompanied by thickening of the skin of palm, sole, torso, and upper limbs (Ahsan et al. 2006b; Mazumder et al. 1998). We instituted a structural protocol by adapting the methods for quantitative assessment of body surface in burn patients. Details of the clinical examination protocol for skin-lesion assessment were previously described (Ahsan et al. 2006b).

At baseline, the study physicians, who were blind to information on the As level in participants’ drinking wells, identified 714 individuals with arsenical skin lesions. A total of 594 cases of skin lesions and a random sample of 1,041 individuals without skin lesions were selected for a study of urinary As metabolites and genetic susceptibility (Ahsan et al. 2007). For the present study, we selected a random sample of 130 cases and 130 noncases from the 156 cases and 422 noncases who were never-smokers, had data on urinary total As and As metabolites, and had blood samples available. After discarding samples that did not have enough serum for the CC16 assay, we included 241 individuals (128 cases and 113 noncases) in the analyses. The project was approved by the Columbia University Institutional Review Board and the Bangladesh Medical Research Council. Verbal informed consent was obtained from all the participants for this study before they were enrolled into the study.

### Sample collection, storage, and processing

#### Water sample collection and As assay

Water samples from the wells the study participants regularly drank from were collected in 50-mL acid-washed tubes following pumping the well for 5 min. These samples were analyzed for As concentration by graphite furnace atomic-absorption (GFAA) with a Hitachi Z-8200 system (Hitachi, Tokyo, Japan) in the Geochemistry Laboratory at Lamont Doherty Earth Observatory of Columbia University. A detailed description of the water-collection procedure has been reported elsewhere (van Geen et al. 2002).

#### Urine sample collection and As assay

Spot urine samples were collected in 50-mL acid-washed tubes and kept in portable coolers with ice packs (carried by the research team) until storage at −20°C at the end of the day. All samples were frozen until shipment on dry ice to Columbia University. Urinary As concentration assays were performed with GFAA using a PerkinElmer Analyst 600 graphite furnace system (PerkinElmer, Wellesley, MA, USA) in the Department of Environmental Health Sciences of Columbia University, as described by Nixon et al. (1991). Levels of As in urine were expressed as micrograms of As per gram creatinine, and creatinine levels were analyzed by a colorimetric Sigma Diagnostics Kit (Sigma Chemical Co., St. Louis, MO, USA).

#### Urinary As metabolites assay

Urinary As metabolites were assayed by inductively coupled plasma-mass spectrometry with dynamic reaction cell (ICP-MS-DRC) coupled to high-performance liquid chromatography (HPLC). ICP-MS-DRC was used as a detector for As metabolites chromatographically separated on an Anion Exchange Column (Hamilton PRP-X100; Hamilton, Reno, NV, USA) with 10 mM ammonium nitrate/ammonium phosphate, pH 9.1, as mobile phase (van Geen et al. 2002). The excellent separation power of HPLC coupled with very low detection limits of ICP-MS-DRC allowed us to detect arseno-choline (AsC), arsenobetaine (AsB), inorganic As (InAs; i.e., AsIII, AsV), total monomethylarsonic acid (MMA), and total dimethylarsinic acid (DMA).

### Blood sample collection and processing

Blood samples were collected in 10-mL vacutainers with silica clot activator and polymer gel separator. Blood samples were stored in a cold box at 4°C immediately after collection. To separate serum from the whole blood, the blood samples were centrifuged 10 min at 4,000 rpm. Separated samples were then stored at −20°C until they were shipped on dry ice to Columbia University for analysis. The serum samples were aliquotted into small plastic tubes and shipped on dry ice to the laboratory at the Catholic University of Louvain for analysis of CC16.

#### CC16 assay

The concentration of CC16 in serum was determined using the assay method described by Hermans et al. (2001). This assay uses the rabbit anti-protein 1 antibody from Dakopatts (Glostrup, Denmark) and the CC16 protein purified in the laboratory as a standard. To avoid interferences by complement, rheumatoid factor, or chylomicrons, sera were prepared by heating at 56^o^C for 30 min and by adding polyethylene glycol (16% vol/vol, 1/1) and trichloroacetic acid (10% vol/vol, 1/40). After overnight precipitation at 4°C, the serum samples were centrifuged (at 3,000 × *g* for 10 min) and CC16 was determined in the supernatants. This assay has a detection limit of 0.5 μg/L and an average analytical recovery of 95%. All laboratory assays for serum CC16 were performed in duplicate at two different and independent dilutions of the samples to achieve maximum accuracy. The within- and between-run coefficient of variation (CV) was between 5 and 10%, and the values were in the normal range (5–20 μg/L for healthy subjects 20–60 years of age) (Shijubo et al. 1999a, 1999b). In epidemiologic studies of biomarkers, CVs up to 15% are often acceptable (Tworoger and Hankinson 2006). The CC16 concentration in serum is in good agreement with levels obtained with a monoclonal antibody–based enzyme-linked immunosorbent assay (ELISA) kit recently developed by Pharmacia (Human Clara Cell Protein ELISA; Pharmacia Biotechnology, Uppsala, Sweden).

### Indices of As exposure and metabolism

#### Cumulative As exposure index

At the baseline interview for each participant, information on daily water consumption and duration of well use was collected for wells used on a regular basis. In addition, similar information was also collected on any other well that a participant used currently (or had used in the past). When the previously used well was within the study area and the As concentration was known, As concentration of the previous well was incorporated in the calculation of the cumulative As exposure index (CAI). Based on well utilization history, we calculated the CAI, which is the sum of the products of the amount of water consumed per day (liters per day) × As concentration in the well(s) (milligrams per liter) × the duration of well use (days) for each well (Ahsan et al. 2006b). The CAI is a good indicator of long-term As exposure (Ahsan et al. 2006a).

#### Arsenic metabolism indices

The percentages of InAs, MMA, and DMA were calculated after subtracting AsC and AsB (i.e., nontoxic dietary sources of As) from the total. An alternative method for describing As metabolite data employs primary (PMI) and secondary (SMI) methylation indices. PMI is the ratio of MMA to InAs, and SMI is the ratio of DMA to MMA.

### Lung function test using spirometry

From the 241 study subjects, we chose a random sample of 40 individuals without skin lesions for lung function testing using spirometry. The tests were repeated at least 3 times to obtain acceptable maneuvers. After accounting for individuals who were unavailable for testing (*n* = 4) or who produced readings of poor quality (*n* = 5), a total of 31 spirometry test results were available for analysis. We used a portable, battery-operated ultrasound transit-time based spirometer (EasyOne; NDD Medical Technologies, Chelmsford MA, USA; and Zurich, Switzerland) for pulmonary function tests. The device is standardized to meet American Thoracic Society guidelines for lung function tests. The EasyOne has been used in a number of research studies in different countries and at a number of teaching hospitals in the United States (Menezes et al. 2005; NDD Medical Technologies 2007; Perez-Pedilla et al. 2006). Although, the EasyOne does not require calibration, we compared its readings among a number of patients and healthy volunteers with readings from a standard spirometer clinically used at the Dhaka Chest Institute in Bangladesh, in collaboration with M. Rashidul Hasan, an expert pulmunologist. Agreement between the spirometry used at the hospital and EasyOne was excellent.

### Statistical analysis

Preliminary analysis involved calculations of frequency distributions, means, and tabular statistics. We performed Student’s *t*-tests for continuous variables between two different groups (e.g., with and without visible skin lesions, lung function tests). The distributions of the outcome and As variables were skewed; therefore, they were log-transformed. Pearson correlation coefficients were used to evaluate the relationship between CC16 and As exposure variables, as well as the relationships of lung function indices with urinary As and As methylation indices. Multivariate linear regression was used to evaluate the associations of As exposure variables and urinary As methylation indices with log-transformed CC16 in all the participants, and was performed separately by skin lesion status. Potential confounders including age, sex, and body mass index (BMI) were included in the multiple linear regression models. A conventional cutpoint (70% of the predictive value) (De et al. 2004) was selected to indicate low and high status of lung function in the subgroup with spirometry data. We conducted *t*-tests to compare levels of urinary As and serum levels of CC16 in participants with low and high lung function. In these analyses, all statistical tests were two-tail tests based on the type I error rate of 0.05. Study participants with missing information on any of the covariates were excluded from regression analyses (*n* = 22). All statistical analyses were conducted using the SAS 8.2 statistical package for Windows (SAS Institute Inc., Cary, NC, USA).

## Results

The average age of the study participants was 37 years (Table 1). Cases with skin lesions were about 6 years older than noncases. Because of our study design, roughly one-half (53%) of the study participants had arsenical skin lesions. Among individuals with skin lesions, 34.3% of male and 35.1% of female participants consumed water containing > 50 μg/L As. The average BMI among the study participants was 20; people with skin lesions had slightly lower BMIs (mean ± SD, 19.4 ± 2.6) than those without lesions (20.4 ± 3.3). As in the overall cohort (Ahsan et al. 2006b), cases with skin lesions had lower body weight than noncases.

The mean As concentration in drinking water was 134 μg/L (Table 1). Individuals with skin lesions consumed water with a significantly higher As concentration (159 μg/L) than those without lesions (105 μg/L). A larger proportion of individuals with skin lesions (37%) than without (25.7%) were also found to drink water > 50 μg/L As concentration. Individuals with skin lesions had significantly higher urinary As (461 μg/g creatinine) than those without (264 μg/g creatinine). Also, the CAI was higher in people with skin lesion (1,815 mg) than in those with no lesions (1,134 mg). The primary As methylation index (MMA/InAs) was also significantly higher among arsenical skin lesion cases (1.01) than noncases (0.84) (*p* < 0.01). Conversely, the secondary methylation index (DMA/MMA) was lower among people with skin lesions (5.6) than among those without (7.8).

Serum CC16 levels were inversely related to urinary As concentrations (*r* = −0.11, *p* = 0.07), although the association did not reach statistical significance at *p* < 0.05 among all individuals. However, among individuals with arsenical lesions, we found significant associations of urinary As (*r* = −0.24, *p* = 0.01) and CAI (*r* = −0.17, *p* = 0.05) with CC16 levels. The association between water As and CC16 was not statistically significant (*r* = −0.12, *p* = 0.15).

Multiple linear regression analyses also revealed no significant association between urinary As and log-transformed CC16 values (β = −0.06, *p* = 0.10) adjusting for age, sex, and BMI in the overall study population (Table 2). Among individuals with skin lesions, we found a significant association of CC16 with urinary As (β = −0.13, *p* = 0.01) and a marginally significant association of CC16 with CAI (β = −0.04, *p* = 0.06).

This analysis revealed a weak negative association between CC16 and the MMA/InAs ratio (β = −0.08, *p* = 0.18) but a marginally significant positive association with the DMA/MMA ratio (β = 0.12, *p* = 0.05) (Table 3). Among individuals without arsenical lesions, the association between CC16 and DMA/MMA ratio was even stronger (β = 0.18, *p* = 0.04), suggesting that As metabolism may be protective of respiratory injury. In addition, our overall data showed a significant association of serum CC16 with MMA% (β = −0.16, *p* = 0.03) but not with DMA% (β = 0.33, *p* = 0.16) in linear regression models after adjusting for age, sex, and BMI.

We also conducted lung function tests by using a spirometer in a small sample (*n* = 31) to ascertain the association between CC16 and clinical lung function. We observed strong inverse associations between urinary As and predictive FEV_1_ (*r* = −0.37, *p* = 0.03; β = −0.017, *p* = 0.03), FVC (*r* = −0.35, *p* = 0.04; β = −0.014, *p* = 0.04) and FEV_1_/ FVC ratio (*r* = −0.36, *p* = 0.04; β = −0.009, *p* = 0.04) among this subset of the study population. Our data show a strong inverse association between FEV_1_/FVC and PMI (*r* = −0.42, *p* < 0.01) (Table 4). Further, we found that individuals (23%) with low (≤ 70) predictive FEV_1_ and FVC values also had marginally significantly lower CC16 compared with those with higher values (> 70; 3.58 and 7.71 μg/L, *p* = 0.05), confirming that individuals with chronic respiratory illness have low levels of serum CC16 (Table 5).

## Discussion

To our knowledge, this is the first study to systematically assess the effects of As from drinking water on respiratory illness using biomarkers of As exposure and lung injury. We found an inverse association between urinary As and serum CC16 level among cases with skin lesions. This could be because individuals with skin lesions are either exposed to a higher levels of As or they are more susceptible to other health effects (especially respiratory effects) of As exposure. The significant associations between serum CC16 and FEV_1_/FVC reinforce the fact that serum CC16 is a biomarker of lung function, and it may be useful in assessing early respiratory damage induced by As, especially among individuals with skin lesions.

Our analyses also showed positive associations of CC16 levels with secondary As methylation index (MMA/DMA), particularly among individuals without skin lesions, suggesting that those with better methylation capacity are less prone to respiratory effects of As. The inverse association of CC16 with MMA% also suggests that individuals with incomplete methylation are more susceptible to the respiratory effect of As. In a large case–control study of As-related skin lesions within the parent cohort study, we also found a dose–response relationship between risk of skin lesions and %MMA (Ahsan et al. 2007).

Although the mechanism of As-induced nonmalignant respiratory illness is not known, several studies have shown that a large amount of As is deposited and stored in the lung, especially in the epithelium (Gerhardsson et al. 1988; Rosenberg 1974; Saady et al. 1989). It is possible that the deposited As in the lung acts like some other metals by enhancing tissue inflammation or increasing pulmonary fibrosis, leading to impaired respiratory function (Nemery 1990). Hotta (1989) suggested that chronic As poisoning renders the respiratory tract more susceptible to infection. von Ehrenstein et al. (2005) suggested that decreased lung function due to As exposure may induce fibrosis and lung impairment. De et al. (2004) suggested an inflammation-mediated immunologic basis of arsenic toxicity in the lung. Some recent articles also suggested a role of oxidative stress in As-induced lung toxicity (Hays et al. 2006; Lantz and Hays 2006).

CC16 is one of the 20 proteins secreted by Clara cells in the lung’s alveolar epithelium, and it plays a major role in protecting the alveolar epithelium from pollutants (Broeckaert et al. 2000). CC16 reflects early lung damage due to chronic environmental exposures (Bernard et al. 1994, 2005; Berthoin et al. 2004; Broeckaert et al. 2000; Gioldassi et al. 2004; Johansson et al. 2005; Lagerkvist et al. 2004; Shijubo et al. 1999a, 1999b). In either chronic inflammation or fibrosis of the lung, alveolar Clara cells are damaged, resulting in a reduced CC16 concentration over time. Recent studies in adults and children have shown significantly lower levels of CC16 in individuals with asthma and rhinitis compared with healthy individuals (Gioldassi et al. 2004; Johansson et al. 2005; Shijubo et al. 1999a, 1999b). Serum CC16 has also been shown to be a reliable measure of lung function in workers exposed to crystalline silica or foundry dust (Bernard et al. 1994; Berthoin et al. 2004; Broeckaert et al. 2000; Lagerkvist et al. 2004). Serum CC16 concentrations are decreased in individuals with compromised lung condition induced by chronic environmental exposures such as cigarette smoking or ozone (Bernard et al. 1994; Berthoin et al. 2004; Lagerkvist et al. 2004). In such conditions Clara cells are damaged by inflammation, which results in decreased production of CC16 and thereby limiting the ability to repair epithelium damage caused by pollutants (Broeckaert and Bernard 2000). The mechanism by which CC16 protects the epithelium is unclear, but some suggest its role as an antioxidant (Broeckaert et al. 2000; Broeckaert and Bernard 2000).

Nonmalignant respiratory effects of As exposure have been investigated in previous studies. Six studies from India and Bangladesh measured prevalence of respiratory symptoms only in people with visible arsenical skin lesions who were exposed to As concentrations > 500 μg/L (De et al. 2004; Mazumder et al. 2000, 2005; Milton et al. 2003; Milton and Rahman 2002; von Ehrenstein et al. 2005). Assessment of respiratory illnesses may not have been completely reliable in these studies. The study participants had visible skin lesions and were from highly As-contaminated areas; this increased chances of interviewer/assessment bias and recall bias. In the present study we evaluated several indices of As exposure, as well as As metabolism, in relation to results of lung function testing and a biomarker of lung function (CC16). The observation that urinary As was inversely associated with FEV_1_, FVC, and FEV_1_/FVC ratio in the 31 persons with no skin lesions confirms the positive associations between respiratory symptoms and As exposure observed in populations with very high concentrations and individuals with skin lesions (De et al. 2004; Mazumder et al. 2000, 2005; Milton et al. 2003; Milton and Rahman 2002; von Ehrenstein et al. 2005). In the present study, study participants were exposed to an average water As concentration of 134 μg/L (range, 0.1 μg/L–761 μg/L), much lower than in previous studies. Our findings on lung function and serum CC16 further provide evidence of lung dysfunction in populations with low to medium levels of As exposure.

Although we observed an association between urinary As and serum CC16, we did not find a strong association with water As concentration. The difference in association for the two measures could be due to the fact that the value of water As, based on a single well, may not reflect the true exposure (if the subject drinks water from multiple wells). In contrast, urinary As reflects the aggregate exposure from all sources. Thus urinary As is considered a better measure of recent total As exposure.

A limitation of our study is that we did not collect detailed clinical information on respiratory illness and that lung function data were only available among a subset of study participants. However, given that the subset was randomly selected, results on lung function observed in the subset of the study population may also apply to all of the study participants. It is also unlikely that the observed association is due to other factors that may influence serum CC16 concentrations. There has been no evidence that As exposure from drinking water is related to factors such as exposure to ozone, occupational exposure to nitric oxides, or asbestos that may influence serum concentrations of CC16. In fact, > 95% of the population in our study area use biofuels for cooking. Chen et al. (2007) reported no apparent association between well As concentration and occupation or indicators of socioeconomic status (SES) including educational status and land ownership. The distribution of occupation of the parent cohort study participants was 53% homemakers, 10% farmers or agriculture labors, 18% small business, 10% workers in textile or dyes, and 10% unemployed. In addition, controlling for indicators of SES, including educational attainment and occupations, did not change the effect estimates appreciably (data not shown).

Several studies have shown that smokers with As exposure are at a higher risk of developing respiratory illness (Mazumder et al. 2005; von Ehrenstein et al. 2005). Our findings further suggest that the effect of As exposure on respiratory illness may be significant among nonsmokers, because we restricted the study to only never-smokers. We have also found that smoking plays an additive role in As-induced respiratory illnesses (Parvez F, Chen Y, Joseph HG, Brandt-Rauf PW, Argos M, Slavkovich V, Islam T, Hassan R, Balac O, and Ahsan H, unpublished data). Hays et al. (2006) has reported a synergistic effect of As and cigarette smoking to increase DNA oxidation in the lung.

Presently, invasive procedures for detecting respiratory illnesses, such as bronchoscopy or bronchoalveolar lavage techniques, are not suitable for large-scale population studies in rural Bangladesh. Also, self-reported symptoms or lung function tests usually detect disease with relatively late-stage lung damage. In contrast, an appropriate biological marker such as CC16 can be used to detect respiratory illnesses at an early stage and it is easy to use, making it especially attractive for tracking respiratory damage from As or other exposure in populations such as in Bangladesh.

In conclusion, we observed an inverse association of serum CC16 with As exposure and a positive association with As methylation capacity in this Bangladeshi population. These associations differed by participants’ skin lesion status. We infer that the methylation of InAs to DMA is protective against respiratory effects of As, because those with a higher ratio of DMA/MMA appeared to be at decreased risk for lung dysfunction. We also observed a deleterious effect of urinary As on lung function, as assessed by FEV_1_ and FVC. These novel findings need to be confirmed in future larger studies.

## Figures and Tables

**Table 1:** Demographic, CC16, and As-related variables for the study participants, by skin lesion status.

	Overall (*n* = 241)	Subjects with skin lesions (*n* = 128)	Subjects without skin lesions (*n* = 113)	*p*-Value^a^
Male (%)	32.3	50.0	12.0	< 0.01
CC16 (μg/L)	6.39 ± 3.1	6.42 ± 3.3	6.36 ± 2.9	0.87
Age (years)	37 ± 9.5	39.7 ± 9.8	33.8 ± 8.1	< 0.01
BMI	19.9 ± 3.0	19.4 ± 2.6	20.4 ± 3.3	0.01
Water As (μg/L)	134 ± 153.8	159 ± 181.1	105 ± 109.2	< 0.01
Urinary As (μg/L)	189 ± 250.2	213 ± 251.3	162 ± 182.3	0.10
Creatine (μg/L)	57 ± 45.8	51 ± 41.5	64 ± 49.6	0.03
Urinary creatinine-adjusted As (μg/g creatinine)	368 ± 367.6	461 ± 451.4	264 ± 194.5	< 0.01
Cumulative As (mg)	1,499 ± 2,436	1,815 ± 2,763	1,134 ± 1,941	0.02
MMA/InAs	0.932 ± 0.5	1.01 ± 0.48	0.84 ± 0.52	0.01
DMA/MMA	6.6 ± 3.0	5.6 ± 2.6	7.8 ± 4.6	< 0.01

Values shown are mean ± SD except where indicated.

a *p*-Value from *t*-test or chi-squared tests.

**Table 2:** Linear regression analyses for the effects of different measures of As exposure on serum CC16 levels among all participants, as well as stratified by skin lesion status.

	Log-transformed CC16					
	Overall model (*n* = 238)		Subjects with skin lesions (*n* = 126)		Subjects without skin lesions (*n* = 112)	
Log-transformed measure	Parameter estimates^a^	*p*-Value	Parameter estimates^a^	*p*-Value	Parameter estimates^a^	*p*-Value
Water As (μg/L)	−0.01841	0.31	−0.03853	0.15	0.00564	0.82
Urinary creatinine-adjusted As (μg/g creatinine)	−0.06070	0.10	−0.13462	0.01	0.03476	0.54
Cumulative As (mg)	−0.01745	0.29	−0.04527	0.06	0.01547	0.52

a Parameter estimates were adjusted for age, sex, and BMI.

**Table 3:** Linear regression analyses for the effects of urinary As metabolites on serum CC16 levels among all participants as well as stratified by skin lesions status.

	Log transformed CC16					
	Model (*n* = 238)		Subjects with skin lesions (*n* = 126)		Subjects without skin lesions (*n* = 112)	
Log-transformed measure	Parameter estimate^a^	*p*-Value	Parameter estimate^a^	*p*-Value	Parameter estimate^a^	*p*-Value
MMA:InAs	−0.08218	0.18	−0.07449	0.47	−0.08224	0.30
DMA:MMA	0.12532	0.05	0.04883	0.62	0.18238	0.04

a Adjusted for age, sex, BMI, and log-transformed water As concentration.

**Table 4:** Pearson’s correlation coefficients for the associations between urinary As and its metabolites and spirometric measures (predictive FEV_1_ and FVC values) of lung functions (*n* = 31).

Spirometry measures	Urinary As *r* (*p*)	MMA:InAs *r* (*p*)	DMA:MMA *r* (*p*)
FEV_1_	−0.37 (0.03)	−0.21 (0.20)	0.30 (0.09)
FVC	−0.35 (0.04)	−0.05 (0.70)	0.27 (0.14)
FEV_1_/FVC	−0.36 (0.04)	−0.42 (0.01)	0.29 (0.10)

**Table 5:** Serum CC16 concentrations by predictive FEV_1_ and FVC values (*n* = 31).

Spirometry measures	Predictive value	Percent (*n*)	Mean urinary As (μg/L)	Mean CC16 (μg/L)	*p*-Value^a^
FEV_1_	≤ 70	23 (7)	514.42	3.58	0.05
	> 70	77 (24)	329.08	7.71	
FVC	≤ 70	26 (8)	460.5	3.50	0.02
	> 70	74 (23)	339.78	7.92	

a Between mean differences of CC16 concentration.
